# Neuroprotective Mechanisms of *Lycium barbarum* Polysaccharides Against Ischemic Insults by Regulating NR2B and NR2A Containing NMDA Receptor Signaling Pathways

**DOI:** 10.3389/fncel.2017.00288

**Published:** 2017-09-27

**Authors:** Zhongshan Shi, Lihui Zhu, Tingting Li, Xiaoya Tang, Yonghui Xiang, Xinjia Han, Luoxing Xia, Ling Zeng, Junhua Nie, Yongxia Huang, Chi Kwan Tsang, Ying Wang, Zhigang Lei, Zaocheng Xu, Kwok-fai So, Yiwen Ruan

**Affiliations:** ^1^GHM Institute of CNS Regeneration (GHMICR), Jinan University, Guangzhou, China; ^2^Co-innovation Center of Neuroregeneration, Nantong University, Nantong, China; ^3^Department of Anatomy, Jinan University School of Medicine, Guangzhou, China; ^4^Ministry of Education CNS Regeneration International Collaborative Laboratory, Jinan University, Guangzhou, China; ^5^Department of Ophthalmology and State Key Laboratory of Brain and Cognitive Sciences, The University of Hong Kong, Hong Kong, China; ^6^Clinical Neuroscience Institute, The First Affiliated Hospital, Jinan University, Guangzhou, China; ^7^Key Laboratory of South China Agricultural Plant Molecular Analysis and Genetic Improvement, Provincial Key Laboratory of Applied Botany, South China Botanical Garden, Chinese Academy of Sciences, Guangzhou, China; ^8^Department of Anatomy and Cell Biology, Indiana University School of Medicine, Indianapolis, IN, United States

**Keywords:** ischemia, *Lycium barbarum* polysaccharides, excitotoxicity, apoptosis, NR2B, NR2A

## Abstract

Glutamate excitotoxicity plays an important role in neuronal death after ischemia. However, all clinical trials using glutamate receptor inhibitors have failed. This may be related to the evidence that activation of different subunit of NMDA receptor will induce different effects. Many studies have shown that activation of the intrasynaptic NR2A subunit will stimulate survival signaling pathways, whereas upregulation of extrasynaptic NR2B will trigger apoptotic pathways. A *Lycium barbarum* polysaccharide (LBP) is a mixed compound extracted from *Lycium barbarum* fruit. Recent studies have shown that LBP protects neurons against ischemic injury by anti-oxidative effects. Here we first reported that the effect of LBP against ischemic injury can be achieved by regulating NR2B and NR2A signaling pathways. By *in vivo* study, we found LBP substantially reduced CA1 neurons from death after transient global ischemia and ameliorated memory deficit in ischemic rats. By *in vitro* study, we further confirmed that LBP increased the viability of primary cultured cortical neurons when exposed to oxygen-glucose deprivation (OGD) for 4 h. Importantly, we found that LBP antagonized increase in expression of major proteins in the NR2B signal pathway including NR2B, nNOS, Bcl-2-associated death promoter (BAD), cytochrome C (cytC) and cleaved caspase-3, and also reduced ROS level, calcium influx and mitochondrial permeability after 4 h OGD. In addition, LBP prevented the downregulation in the expression of NR2A, pAkt and pCREB, which are important cell survival pathway components. Furthermore, LBP attenuated the effects of a NR2B co-agonist and NR2A inhibitor on cell mortality under OGD conditions. Taken together, our results demonstrated that LBP is neuroprotective against ischemic injury by its dual roles in activation of NR2A and inhibition of NR2B signaling pathways, which suggests that LBP may be a superior therapeutic candidate for targeting glutamate excitotoxicity for the treatment of ischemic stroke.

## Introduction

Glutamate excitotoxicity is a major factor in ischemia-induced neuronal death (Nishizawa, 2001). Excess release of glutamate from presynaptic membranes induced by ischemia overactivates glutamate receptors leading to a series of events including intracellular calcium overload, excessive ROS production and mitochondrial stress and finally neuronal death (Benveniste et al., 1984; Lipton and Rosenberg, 1994). However, based on accumulating evidence in the literature, all clinical trials using glutamate receptor inhibitors have failed (Koh and Choi, 1991; Morris et al., 1999; Albers et al., 2001; Ikonomidou and Turski, 2002) although some of the inhibitors reduced ischemic damage in animal experiments (Lin et al., 1993; Reyes et al., 1998; Cai, 2006). It is known that glutamate receptors play important roles in maintaining physiological functions such as excitatory signal transduction, learning and memory (Mayer and Westbrook, 1987; Newcomer et al., 2000). Therefore, a more promising strategy for treating ischemic stroke may be through selectively blocking excitotoxicity while preserving important physiological aspects of glutamate receptor subunit function (Cho et al., 2010).

Previous studies have identified three NMDAR subunits: NR1, NR2 (A–D) and NR3 (A,B). Functional NMDARs are heterotetramers composed of two glycine–biding NR1 subunits and two glutamate-binding NR2 subunits, whereas NR1/NR3 heterotetramers can be combined by glycine (Chatterton et al., 2002; Mayer and Armstrong, 2004; Paoletti, 2011). Different NR2-containing receptors (NR1/NR2A, or NR1/NR2B heterotetramers) exhibit different biophysical and pharmacological properties (Cull-Candy and Leszkiewicz, 2004; Furukawa et al., 2005; Chen and Wyllie, 2006). NR2A subunits are primarily located at intrasynaptic sites, whereas NR2B subunits are predominantly located at extrasynaptic sites (Stocca and Vicini, 1998; Rumbaugh and Vicini, 1999; Tovar and Westbrook, 1999; Traynelis et al., 2010). Functionally, NR2A subunits play a neuroprotective role by activating cellular CREB or Akt pathways (Hardingham and Bading, 2010; Luo et al., 2011; Lai et al., 2014). Activation of NR2A will induce phosphorylation of CREB which is associated with BDNF expression and contributes to neuronal survival (Chen et al., 2008). It has been reported that some drugs may be neuroprotective against ischemia via enhancing CREB activity (Raval et al., 2009; Zhang et al., 2010). Conversely, activation of the extrasynaptic NR2B subunit will trigger apoptotic pathways by increasing ROS levels and prohibiting CREB expression (Léveillé et al., 2008; Hardingham and Bading, 2010; Gladding and Raymond, 2011). When animals experience ischemic insult, nNOS will translocate to the cell membrane to form the NR2B-PSD95-nNOS complex that activates nNOS to produce more NO and causes severe neuronal injury. Some agents have recently been tested in the rat MCAO model and stroke primates showing that disruption of nNOS-PSD95 or NR2B-PSD95 interaction reduced infarct area in ischemic models (Zhou et al., 2010; Cook et al., 2012). Whether both NR2A and NR2B subunits could be simultaneously regulated to achieve neuroprotection by pharmacological drugs remains unknown.

*Lycium barbarum* (*Gouqi* or wolfberry) is well known as a traditional Chinese medicine and healthy food supplement in China and other countries (Amagase et al., 2009). *Lycium barbarum* polysaccharide (LBP) is a mixed compound extracted from the fruits of *Guoqi*, composing primarily of rhamnose, arabinose, xylose, galactose, mannose and galacturonic acid (Zou et al., 2010). Recent years, many studies have investigated LBP’s structures, bioactive components and degradation. Studies have found that the water-soluble LBP containing 3.75% proteins has a β-D-(1 → 6)-galactan as a backbone with highly branched polysaccharide (Wang Z. et al., 2014; Yuan et al., 2016). A new study reported that a p-LBP was isolated and purified from LBP-and p-LBP was a homogeneous heteropolysaccharide as a pectin molecule with an average molecular weight of 64 kDa, approximately 87 nm hollow sphere in 0.05 mol/L sodium sulfate solution (Liu et al., 2016). Many studies have shown that LBP has strong anti-oxidative effects in diverse injury types, including doxorubicin-induced cardiotoxicity (Xin et al., 2011) and oxidative liver injury (Wu et al., 2010; Xiao et al., 2012). In addition, some studies showed that LBP plays beneficial roles in aging (Tang and He, 2013), hypoglycemia (Zhu et al., 2013) and anti-radiation or chemotherapy damages (Gong et al., 2005). Concerning nervous system damage, LBP protected ganglion cells against acute ocular hypertension (Mi et al., 2012), partial optic transection injuries (Li et al., 2013) and retinal ischemia/reperfusion injury (Li et al., 2011). Furthermore, LBP can prevent cortical neurons from damage against ischemic insults through anti-oxidative and anti-apoptotic mechanisms (Rui et al., 2012; Wang T. et al., 2014).

LBP appears to have broad benefits in a variety of injury types by regulating different pathways. Based on the idea that different NMDA receptor subunits have distinct functions, we hypothesized that LBP exhibits its neuroprotective effect against ischemic injury by regulating NR2A and NR2B signaling pathways. The present study investigated the underlying mechanisms of LBP-induced neuroprotection in *in vivo* ischemia and *in vitro* oxygen-glucose deprivation (OGD) models and unraveled that indeed both NR2A and NR2B receptor signaling pathways play crucial role in the actions of LBP.

## Materials and Methods

### Animals and Groups

Adult male Wister rats (220–300 g) were obtained from the Animal Experiment Center of Southern Medical University (Guangzhou, China). Animals were housed and cared for in accordance with the Guide for the Care and Use of Laboratory Animals of the National Institutes of Health. All experimental protocols involving rats were approved by the ethical committee of Jinan University and were carried out in accordance with approved guidelines and regulations. All efforts were made to minimize the number of animals used and their suffering.

Animals were randomly assigned to five groups (*n* = 10 per group): a sham group, vehicle group (fed PBS for 1 week before and after four-vessel occlusion (4-VO) ischemia), Pre-LBP group (fed LBP for 1 week before ischemia followed by feeding PBS for 1 week), the Post-LBP group (fed PBS for 1 week before ischemia followed by feeding with LBP for 1 week), and LBP-LBP group (fed LBP for 1 week before and after ischemia).

### Administration of LBP *In Vivo*

LBP was purified from *Lycium barbarum*, which was harvested in Ningxia province of China. The preparation for LBP extracts was performed as reported previously (Yu et al., 2005). LBP was dissolved in PBS at dose of 4 mg/mL according to previous study (Li et al., 2013). LBP (20 mg/kg body weight) was administrated intragastrically with an oral gavage needle to the rats according to methods used in previous research (Wang T. et al., 2014). Standard chow diet and tap water were freely available to all rats.

### Transient Global Ischemia

Transient global ischemia was induced by using the 4-VO method with minor modifications (Pulsinelli et al., 1982; Han et al., 2016). Briefly, rats were anesthetized by intraperitoneal (i.p.) injection of 400 mg/kg chloral hydrate (Fluka, Buchs, Switzerland). Body temperature was maintained at 37.0 ± 0.2°C during surgery using heating pads. The bilateral vertebral arteries were exposed and permanently electrocauterized. Both common carotid arteries (CCAs) were occluded with carotid artery clips for 15 min, and blood flow was monitored at a point in the skull (bregma: −2.0 mm, midline: 5.0 mm) with a probe connecting a laser-Doppler flowmetry device (MoorVMS-LDF2, Moor Instruments, Devon, UK) before, during and after ischemia. The ischemic model was considered successful when the cerebral blood flow decreased to 10%–15% of pre-ischemic levels and returned to the baseline (100 ± 10%) after release of the carotid artery clips. Sham animals underwent all aspects of handling and surgery without occlusion of the vertebral and CCAs.

### Morris Water Maze

One week after reperfusion, the mice were trained to perform in the Morris water maze navigation task. A circular pool (1.2 m diameter and 50 cm depth) was filled with water (maintained at 23 ± 2°C). A 10-cm platform was placed 1.5 cm underneath the water surface (to hide its visibility) in one quadrant of the pool. The navigation task was performed in the present study as previously described (Jing et al., 2015). Briefly, rats were first adapted to swimming for 2 min the day before testing. Over the following four consecutive days, rats underwent learning trials to escape water by finding the invisible platform. The maximum time for each rat to swim in the pool was 60 s. If the animal did not find the platform within 60 s, the rat was gently guided to the platform and left on it for 15 s. Each rat underwent four trials at four starting positions in four quadrants each day with an interval of 15 s. The platform was removed on the fifth day, and the rats were allowed to navigate in the pool for 60 s. The escape latency to find the platform in the first 4 days and the duration of swimming in the platform quadrant at the fifth day were recorded. All trial information was collected automatically by a video camera linked to an animal behavioral recording system (Ethovison XT, Noldus Information Technology Co, Netherland).

### Tissue Preparation for Hematoxylin-Eosin (HE) Staining

The day after finishing behavior assessment, the rats were anesthetized with 10% chloral hydrate (400 mg/kg, i.p) and then transcardially perfused with 0.9% NaCl solution followed by 4% paraformaldehyde in 0.1 M phosphate buffer (PB, pH 7.4). Brains were removed and post-fixed in the same fixative at 4°C for 24 h. Brain blocks containing the hippocampus were processed for dehydration with an increasing alcohol gradient and three xylene clearing steps with an Automated Tissue Processor (LYNX II, Hatfield, PA, USA). The blocks were embedded with paraffin in an embedding machine (Thermo Scientific HistoStar, Kalamazoo, MI, USA) and sectioned at 10 μm thickness with a paraffin microtome (Leica, RM2235, Wetzelar, Germany). Sections were mounted on slides for Hematoxylin-Eosin (HE) staining by two individuals blinded to the treatment groups.

### Quantitative Analysis of Hippocampal CA1 Neuron Damage

At least five animals in each group were assessed for neuronal injury. Six to nine sections with typical hippocampal structures in each animal were photographed under a microscope (Leica, CTR 6000) using 10× objective with 10× eyepiece field. The degree of neuronal damage in the CA1 area was evaluated based on extent of neuron death (Pulsinelli and Brierley, 1979); level 0 (no damage), level 0.5 (<10%), level 1.0 (10%–30%, mild damage), level 1.5 (30%–50%, mild-to-moderate damage), level 2.0 (50%–70%, moderate damage), level 2.5 (70%–90%, moderate-to-severe damage) and level 3.0 (>90%, severe damage). The original quantitative data was analyzed with StatView 5.1 software. Differences were considered significant when *p* < 0.05.

### Primary Cortical Neuron Culture

For primary cortical culture, female Wistar rats (provided by Jinan University animal center) were anesthetized with chloral hydrate (400 mg/kg) at E18 gestation, and embryos extracted from the uterus. The brains of embryos were removed and placed into separate dishes. Next, the cortices were dissected and cut into pieces, followed by digestion with 0.125% trypsin (w/v, Gibco, USA) at 37°C for 30 min. Digestion was stopped by 10% FBS (Gibco). After suspension, centrifugation and filtration, cells were re-suspended in Neurobasal media (Gibco) containing 2% B27 (Gibco), 2 mM glutamine (Gibco), 1 mM sodium pyruvate, 25 μM glutamate, 100 U/ml penicillin and 100 μg/ml streptomycin (Gibco). The cells were then plated on multiwell plates or dishes (Corning, USA) coated with 0.01% (w/v) poly-L-lysine (Sigma, USA) at a density of 10^5^ cells/cm^2^ and placed in a standard incubator (Heal Force, China) maintained at 37°C in 95% air-5% CO_2_. For immunohistochemical detection and fluorescent staining, cells were plated on coverslips in 24-well plates or in dishes in the same density. Half of the medium was changed every 3 days.

### OGD and Reperfusion

After cells were cultured in wells or dishes for 2 weeks, the culture medium was removed and the cells washed three times with deoxygenated (bubbled with N_2_ to remove the residual O_2_ for 30 min) and glucose-free BSS (consisting of 116 NaCl, 5.4 KCl, 0.8 MgSO_4_, 1.0 NaH_2_PO_4_, 26.2 NaHCO_3_, 1.8 CaCl_2_, all in mM). Then, the wells or dishes were placed in an anaerobic chamber (Coy Laboratory, USA) with 5% CO_2_-95% N_2_ at 37°C for a designated period of OGD. OGD was terminated by replacement of normal culture medium. Cells were placed back in a standard incubator for 24 h before being analyzed. A control group was cultured with normal culture medium in the standard incubator.

### *In Vitro* Drug Administration

LBP was dissolved in DDW at a concentration of 100 mg/ml to prepare a stock solution, and the LBP working solution was diluted to 100 mg/l based on a previous study (Ho et al., 2009). D-serine (S4250, Sigma) was first dissolved in DDW as stock solution (200 mM). A stock solution of NVP-AAM077 (P1999, Sigma) was prepared in DMSO (100 μM). Based on other studies (Martin et al., 2003; Tovar et al., 2013), the working concentration of D-serine was 200 μM and NVP-AAM077 was 100 nM.

### Detection of Neural Viability and Mortality

Neural viability was measured using a MTT reduction kit (Sigma, USA). Briefly, primary cultured neurons were exposed to designated periods of OGD. Twenty-four hours after the reperfusion, neurons were incubated with 500 μg/ml MTT in culture medium at 37°C for 4 h. Next, the medium was removed, and 100 μL DMSO was added into each well of 96 well multiplates to dissolve the crystals. The absorbance at 550 nm was measured with a microplate reader (PerkinElmer, USA). Neural death was determined by measuring the concentration of LDH released from the damaged cells with a LDH assay kit (Dojindo, Japan) following manufacturer’s instruction.

### Western Blot Analysis

Cells cultured in dishes were lysed in ice-cold lysis buffer with appropriate protease inhibitors (Roche, USA). Lysed cells in lysis buffer were centrifuged (14,800 rpm, 20 min, 4°C) and supernatant was collected. The supernatant protein concentration was determined using a bicinchoninic acid (BCA) protein assay kit (Pierce, USA) at an absorbance at 562 nm in a microplate reader (PerkinElmer, USA). Protein concentration was then calculated according to the standard curve. Equivalent amounts of protein (40 μg) were separated by sodium dodecyl sulfate-polyacrylamide electrophoresis (SDS-PAGE) using 8%–12% Bis–Tris gels (Beyotime, China). Proteins initially ran through a stacking gel for 30 min at 90 V using an electrophorator (Bio-Rad, USA), and then through a separating gel at 120 V for 60–90 min. Then the proteins were then transferred onto PVDF membranes with a constant current of 250 mA for 60 min. Following a blocking step (0.1% Tween-20, 5% nonfat milk in 0.1% TBS for 1 h, RT), the PVDF membranes were incubated with primary antibodies overnight at 4°C with gentle shaking. The primary antibodies and dilutions used were as follows: nNOS, PSD95, cytC, Bcl-2-associated death promoter (BAD) and GAPDH (1:1000, Abcam, UK); pCREB (Ser133), pAkt (Ser-473) and cleaved caspase-3 (1:1000, Cell Signaling Technology, USA); NR2Aand NR2B (1:1000, Millipore, USA). All antibody dilutions were made using 0.1% TBST with 5% BSA. After three 10 min washes, the membranes were then incubated with HRP conjugated anti-rabbit IgG or anti-mouse IgG (1:1000, Abcam, UK) followed by incubation with ECL reagent (WBKLS001000, Millipore, USA) for 5 min, and detected with an imaging system (Alliance 6.7, UVITEC Limited, UK).

### Immunofluorescence Staining

Cells cultured on coverslips were fixed with 4% paraformaldehyde for 10 min at room temperature. After three washes with 0.01 M PBS, non-specific proteins were blocked with a blocking buffer containing 5% (v/v) normal goat serum (Sigma), 3% (w/v) BSA (Sigma) and 0.05% (v/v) Triton X-100 (Sigma) in PBS at RT for 30 min. Next, the cells on coverslips were incubated with primary antibodies (MAP2, 1:1000, Millipore or GFAP, 1:3000, Millipore) overnight at 4°C. Following three rinses with PBS, the coverslips were incubated with an appropriate fluorescence-conjugated secondary antibody (1:1000, Jackson, USA) at RT for 1 h followed by three rinses. Finally, the coverslips were mounted with Antifade Mounting Medium (Vector, USA) with or without dihydrochloride (DAPI, 1:1000, Sigma, USA). Images were acquired with a fluorescence microscope (Leica, Germany).

### Fluoro JADE B Staining

Neural damage was identified using Fluoro JADE B (FJB, Sigma, USA) following a protocol modified from the manufacturer’s instructions. Briefly, cells cultured on coverslips were immersed in 80% ethanol containing 1% NaOH for 2 mins, and then immersed in 70% ethanol followed by a 1 min rinse with doubly distilled H_2_O (DDW). Next, the cells were treated with 0.06% KMnO_4_ at RT (shaking) for 15 min. After a 1 min rinse with DDW, the cells were incubated with FJB (0.0004%) for 5 min and then rinsed three times with DDW. The coverslips were mounted with 80% glycerin. Photos showing FJB labeling were taken with a fluorescence microscope (Leica, Germany).

### Fluorescent Staining for Measurement of Intracellular Calcium, ROS and Mitochondrial Permeability in Living Cells

Primary cortical neurons were cultured on coverslips in petri dishes. After different experimental processes, the following assessments were performed.

#### Measurement of Intracellular Calcium

Cells were washed with Hank’s solution three times then incubated with 10 μM Fluo-4/AM (Dojindo, Japan) for 30 min at 37°C in the dark followed by three washes with Hank’s solution.

#### Measurement of ROS Level

Intracellular ROS was detected by a molecular probe (CellROX Green reagent, Life Technologies, Camarillo, CA, USA). Briefly, cells were incubated with the reagent (5 μM) for 30 min followed by three washes with PBS.

#### Detection of Mitochondrial Permeability

Cells were incubated with rhodamine123 (1 μM) for 10 min, and then rinsed three times with PBS. Following the last rinse, PBS was replaced by culture medium.

Photos showing the density of intracellular calcium, ROS level and mitochondrial permeability of living cells were taken by a fluorescence microscope (Zeiss, Axio, Germany). The fluorescence intensity in images was analyzed by ImageJ software (NIH).

### Quantification

For all *in vitro* quantitative analyses, three to five experiments were performed. The mean values for MTT and LDH assays were measured from three wells in each group in each cell culture. The final value was determined by comparison of the value of LDH or MTT to that of control group (as 100%). For immunofluorescence quantification, 8–10 photos were randomly taken from four to five coverslips in each group by a fluorescence microscope under a 20× objective in each experiment. Two blinded individuals counted the number of MAP2 or GFAP positive cells. Similar processes of quantitative analysis were performed for FJB, intracellular calcium, ROS and mitochondrial permeability. Fluorescent intensity was measured using ImageJ software (version 1.45 s, NIH, USA). For quantitative protein analysis from Western blotting, photos were obtained with an imaging system (Alliance 6.7, UVITEC Limited, UK). Signal intensities of the membranes were analyzed using Quantity One software (Bio-Rad).

### Statistics

All results were expressed as mean ± SEM. All original data were input to StatView (Version 5.0.1) and a one-way analysis of variance (ANOVA) was performed for multiple comparisons followed by *post hoc* Bonferroni/Dunn test. The difference for each comparison was considered statistically significant at *p* < 0.05.

## Results

### LBP Reduced Neuronal Damage and Attenuated Memory Deficits after Global Ischemia *In Vivo*

**Figure 1:**
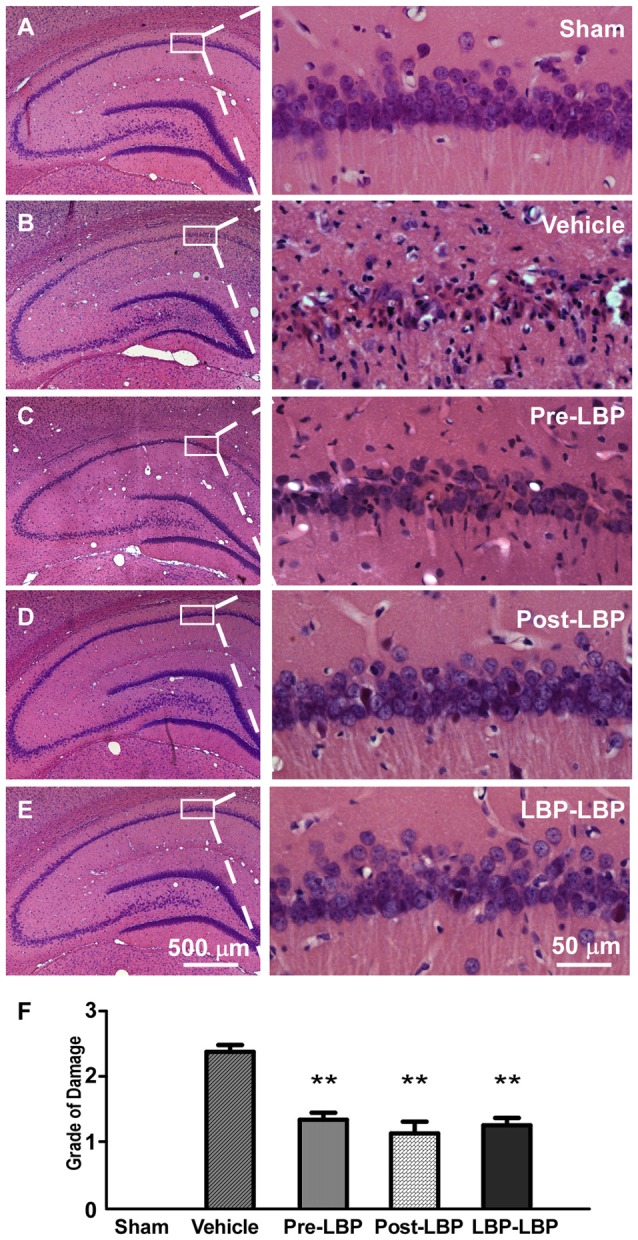
Hematoxylin-Eosin (HE) staining showing effects of *Lycium barbarum* polysaccharide (LBP) treatment on neuronal survival in the CA1 area. Morphological changes of neurons in different treatment groups are shown in representative pictures **(A–E)**. Sham group, without four-vessel occlusion (4-VO) injury; Vehicle group, 4-VO insult only; Pre-LBP group, pretreated with LBP for 1 week before 4-VO; Post-LBP, 4-VO followed by LBP treatment for 1 week; and LBP-LBP, treatment with LBP for 1 week before and after 4-VO. Quantitative analysis is shown in **(F)** (*n* = 5. **P < 0.01).

To test whether LBP is neuroprotective following transient global ischemia *in vivo*, we intragastrically fed rats with LBP for 1 week before or after ischemia, or both. After HE staining of coronal brain sections including the hippocampus, we evaluated the degree of neuronal damage in the CA1 area (damage score from 0 to 3) based on previously established criteria with modification (Pulsinelli and Brierley, 1979). The results showed that approximate 90% of neurons died in the CA1 area in the vehicle group (damage score = 2.4 ± 0.1) at 14 days after ischemia (Figures 1B,F) compared to the sham group (Figures 1A,F; *p* < 0.01). However, with LBP treatment, CA1 neuron morphology improved compared to the vehicle treated group (Figures 1C–E), and the degree of damage of CA1 neurons was significantly reduced in the Pre-LBP, Post-LBP and LBP-LBP groups (damage score =1.3 ± 0.1, 1.1 ± 0.2 and 1.1 ± 0.1 respectively, all *p* < 0.01 vs. vehicle group; Figure 1F).

**Figure 2:**
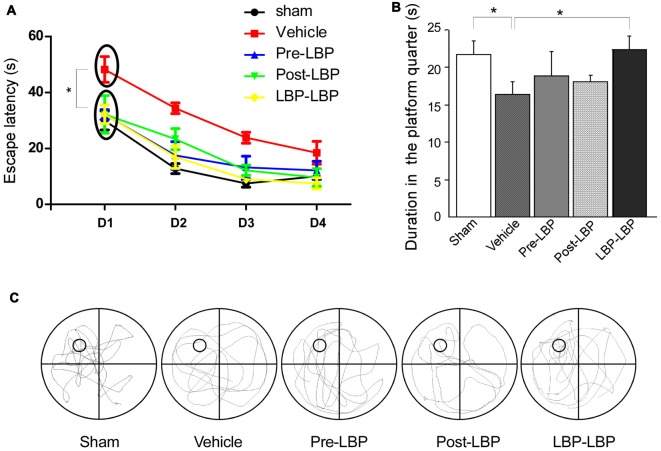
Behavioral analysis in the Morris water maze showing the effects of LBP on ischemia-induced spatial memory deficits. **(A)** Escape latency of rats in different groups during the 4 days’ swimming training. **(B)** Duration in the platform quadrant on the fifth day after the platform was removed. **(C)** Swimming track of rats in different group on the fifth day (*P < 0.05; *n* = 10).

Next we examined the effects of LBP on ischemia-induced memory impairment. Rats received 4 days’ swimming training in Morris water maze from which the escape latency of rats was recorded. The duration in the platform quadrant and swimming track was also measured after removing the platform on the fifth day. As shown in Figure 2A, all groups showed a trend of decreasing escape time with extended training. However, when comparing the mean time during the first 4 days of training period, the vehicle group (31.2 s ± 2.97) took longer time than the sham (15.08 s ± 1.86), Pre-LBP (18.72 s ± 3.38), Post-LBP (19.31 s ± 3.77) and LBP-LBP (16.38 s ± 2.38) groups (all, *p* < 0.05, Figure 2A). For swimming duration in the platform quadrant at day 5 when the platform was removed, the LBP-LBP group (22.1 s ± 1.8) swam significantly longer than the vehicle group (15.6 s ± 2.5, *p* < 0.05, Figure 2B), and performed similarly to the sham group (21.8 s ± 1.7, *p* > 0.05, Figure 2B). Figure 2C illustrates the rats’ movement track at day 5, from which more track lines in the platform quadrant were found in the sham, Pre-LBP, Post- LBP and LBP-LBP groups but less in the vehicle group.

### LBP Enhanced the Viability of Cortical Neurons Exposed to OGD 4 h *In Vitro*

**Figure 3:**
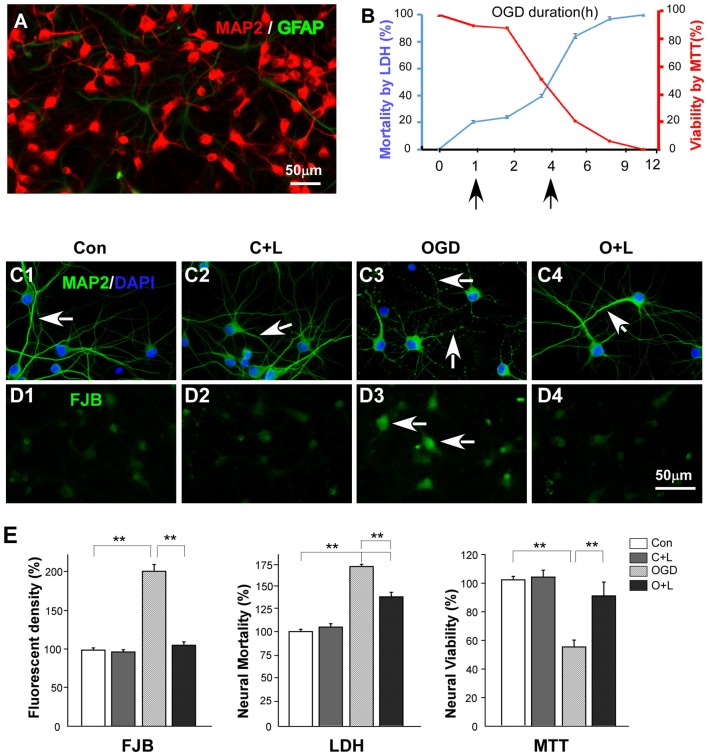
Effects of LBP on neuronal viability and mortality. **(A)** Immunochemistry staining showing that the majority of the cultured cells are neurons (MAP2 positive cells, red) with few astrocytes (GFAP positive cells, green). **(B)** Two slopes showing the relationship between neuronal viability (red slope), death (blue slope) and oxygen-glucose deprivation (OGD) duration. **(C)** Immunochemistry staining showing effects of LBP treatment on morphological changes of neurons exposed to 4 h OGD. Neurons exhibited features of beaded dendrites in the OGD group (**C3**, arrows). However, LBP recovered normal dendritic morphology (**C4**, arrow). **(D)** Fluoro JADE B (FJB) staining (injury marker) showing strong fluorescent labeling in most neurons when exposed to OGD for 4 h (**D3**, arrows). However, LBP treatment reduced the staining intensity (**D4**, arrow). **(E)** Quantitative results showing effects of LBP treatment on neuronal injury (FJB staining), mortality and viability when exposed to 4 h OGD (**P < 0.01). The scale bar in **(D4)** is equal to **(C1–C4)** and **(D1–D3)**.

We carried out *in vitro* experiments to reveal mechanisms of neuroprotection of LBP underlying OGD. Based on findings that there is heterogeneity of hippocampal neurons in response to ischemia such as highly vulnerable CA1 neurons and resistant CA3 neurons, and cortical neurons in the frontal and parietal cortex are more evenly vulnerable to ischemia (Pulsinelli et al., 1982) we cultured cortical cells for 7 days and identified neurons (MAP2) and astrocytes (GFAP) using immunofluorescent staining. Approximately 95% of cells were neurons and 5% astrocytes (Figure 3A). Next, we investigated the relationship between OGD duration (from 1 h to 12 h) and neuronal death. Our results showed that 20% of neurons died when exposed to OGD for 1 h, whereas 50% of neurons were lost after 4 h OGD (Figure 3B). We then detected the protective effect of LBP on cultured neurons exposed to 4 h OGD. Figure 3C shows the effects of LBP on cortical neuron morphological changes. OGD caused neuronal dendrites to become varicose (Figure 3C3, arrows). However, LBP treatment prevented dendritic degeneration (Figure 3C4) and smooth dendrites were found similar to that of control (Figure 3C1) or control + LBP (Figure 3C2) groups. In addition, we detected neural injury with FJB staining (Schmued and Hopkins, 2000). When exposed to OGD for 4 h, strong FJB staining was evident in injured neurons (Figure 3D3, arrow). However, these injury signals were diminished in OGD+LBP neurons (Figure 3D4) with similar weak staining to that observed in control or control + LBP groups (Figures 3D1,D2). Quantitative analysis showed that the density of FJB positive cells increased to 205.2% ± 10.1 after 4 h exposure to OGD (*P* < 0.01 vs. control and Con-LBP groups, Figure 3E). However, value dropped to normal levels (107.9 ± 4.9%, *P* < 0.01 vs. OGD but *P* > 0.05 vs. control and Con-LBP groups) with LBP treatment. Consistent with FJB detection, neuron mortality significantly increased to 168.9% ± 2.3 after 4 h exposure to OGD (*P* < 0.01 vs. control and Con-LBP groups, Figure 3E), whereas it decreased to 137.4% ± 4.0 after the administration of LBP (*P* < 0.01 vs. OGD group). Furthermore, cell viability reduced to 54.2% ± 4.3 in the 4 h OGD group (*P* < 0.01, vs. control and Con-LBP groups, Figure 3E) but increased to 90.2% ± 9.6 after LBP treatment (*P* < 0.01, vs. OGD group).

### Neuroprotective Effects of LBP Was Mediated by Blocking NR2B Signaling Pathway

**Figure 4:**
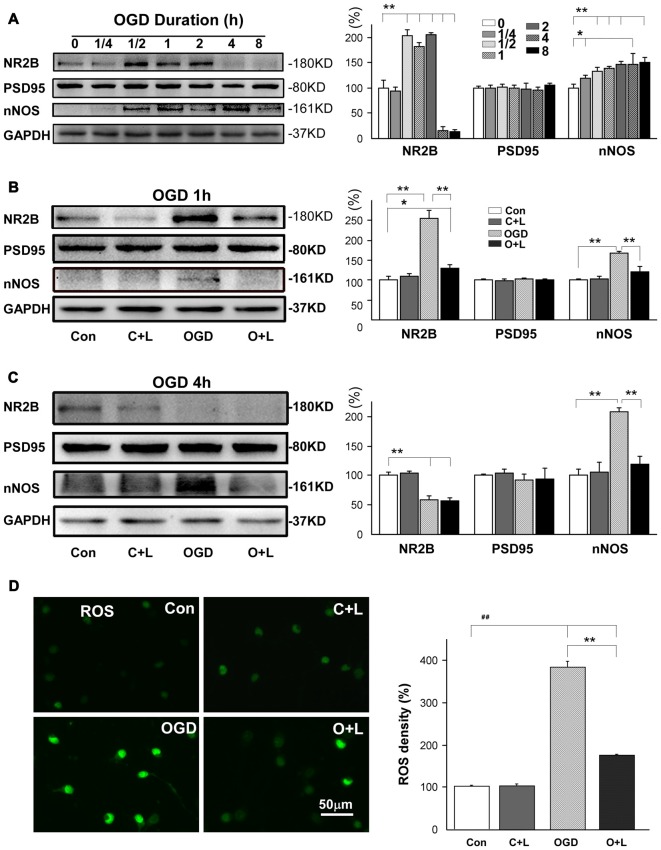
Effects of LBP on the expression of NR2B, PSD95, nNOS and production of ROS after exposed to 1 h or 4 h OGD. **(A)** Expression of NR2B, PSD95 and nNOS by neurons in response to different durations of OGD. **(B)** Administration of LBP blocked the increase in expression of NR2B and nNOS when neurons were exposed to OGD for 1 h. **(C)** LBP application antagonized the increase in nNOS expression, but did not further inhibit NR2B when NR2B level was reduced after 4 h OGD. **(D)** LBP application blocked the increase in ROS production 4 h OGD exposure (*P < 0.05; **P < 0.01; ^##^*P* < 0.01).

After confirming the neuroprotective effects of LBP *in vitro*, we next investigated whether LBP exhibited neuroprotection by blocking NR2B signaling pathway. We detected major proteins in the NR2B signaling pathway. First, we studied the expressions of NR2B, PSD95 and nNOS in different durations of OGD. We found that NR2B expression dramatically increased to approximate twice that of the control level after exposure to OGD from 30 min to 2 h, followed by a marked decrease to 16% ± 4 and 14% ± 2 after 4 h and 8 h OGD duration (all *P* < 0.01 vs. control, Figure 4A). Expression of PSD95 did not show obvious changes with different OGD durations ranging from 15 min to 8 h (*p* > 0.05 vs. control), however, nNOS expression gradually increased as OGD duration increased from 15 min to 8 h (all *P* < 0.05 or *P* < 0.01 vs. control; Figure 4A). Based on these results, we targeted mild injury (OGD for 1 h) and severe injury (OGD for 4 h) to test how LBP affected these protein expressions. With 1 h exposure to OGD, LBP antagonized the increased expression of NR2B from 256% ± 12 to 129% ± 5 and nNOS from 168% ± 2 to 119% ± 9 (both *P* < 0.01 vs. OGD group, Figure 4B). However, LBP did not affect the expression of PSD95 at this time point. With 4 h exposure to OGD and expression of NR2B dropped to 59% ± 4, LBP did not significantly alter the expression of NR2B (56% ± 4; Figure 4C). Likewise, LBP did not alter PSD95 expression at this time point (*P* > 0.05 vs. control). However, LBP markedly reversed the increase in nNOS expression from 208.7% ± 4 to control levels (119.0% ± 8, *P* < 0.01 vs. OGD group and *P* > 0.05 vs. control). We further found that ROS level significantly increased to 302.1% ± 13.5 (*P* < 0.01 vs. control) in neurons exposed to OGD for 4 h, and this increase was blocked by LBP (131.7% ± 11.2, *p* < 0.01 vs. OGD only, Figure 4D).

**Figure 5:**
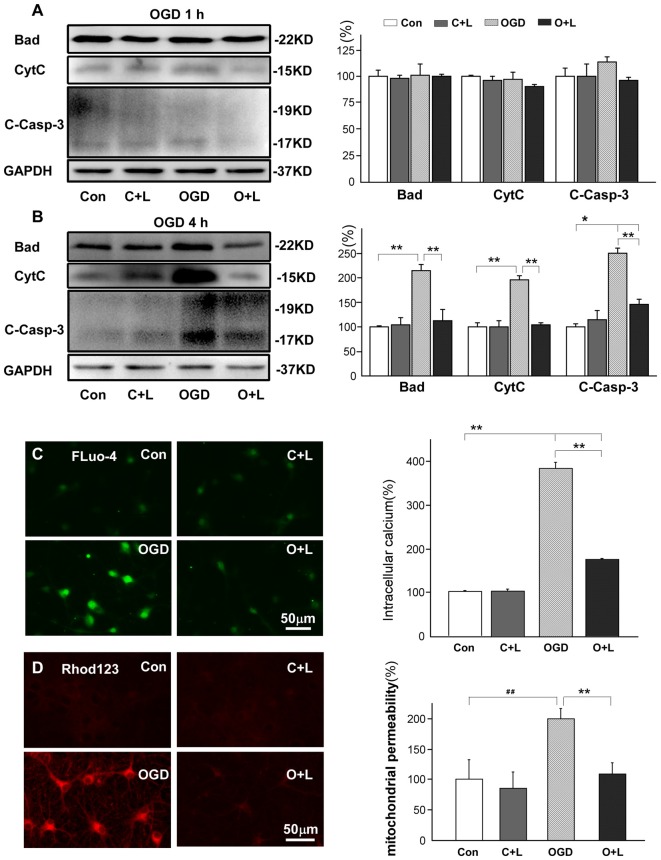
Effects of LBP on expression of Bcl-2-associated death promoter (BAD), cytochrome C (cytC) and cleaved caspase-3, and intracellular calcium and mitochondrial permeability after 4 h OGD exposure. **(A)** LBP did not affect expression of Bad, cytC, or cleaved caspase-3 when these neuronal protein levels stabilized after 1 h OGD. However, in response to 4 h OGD, application of LBP significantly blocked the increase in expression of Bad, cytC and cleaved caspase-3 **(B)**, antagonized the enhancement in intracellular calcium (Fluo-4) **(C)**, and reduced mitochondrial permeability that was detected by Rhodamine 123 (Rohd 123, **D**; *P < 0.05; **P < 0.01; ^##^*P* < 0.01).

It has been reported that nNOS mediates mitochondrial injury (Yao et al., 2012) and subsequently initiates the pro-apoptotic caspase cascade via BAD (Adachi and Imai, 2002), cytochrome C (cytC) and excessive Ca^2+^ influx (Helmreich, 2001; Ito et al., 2013). Therefore, we further investigated whether LBP reduced mitochondria injury and apoptosis. Our results showed that 1 h OGD did not significantly influence expression of Bad, cytC or cleaved caspase-3, and LBP also had no obvious effects on the expression of these proteins (Figure 5A). However, when exposed to OGD for 4 h, the protein level of Bad, cytC and cleaved caspase-3 in neurons increased to 215% ± 7, 196% ± 4 and 251% ± 6 respectively (*p* < 0.01 vs. control, Figure 5B). In addition, LBP significantly reduced Bad, cytC and cleaved caspase-3 expression to 112% ± 13 or 102% ± 2, or 146% ± 5 (all *p* < 0.01 vs. OGD group; Figure 5B). The intracellular concentration of calcium (measured by fluo-4) of cortical neurons increased approximate 4-fold (385% ± 14) at 24 h after exposure to OGD for 4 h (*p* < 0.01 vs. control, Figure 5C). However, LBP dramatically reduced this value to 175% ± 4 (*p* < 0.01 vs. OGD 4 h). The mitochondrial permeability (detected by Rhodamine123) increased to twice that of controls (199.5% ± 3; *p* < 0.01 vs. control, Figure 5D). However, LBP notably reduced the signal density to 109% ± 6 (*p* < 0.01 vs. OGD 4 h), similar to control level (*p* > 0.05 vs. control).

### Neuroprotective Effects of LBP Was also Mediated by Activating NR2A-Akt-CREB Pathways

**Figure 6:**
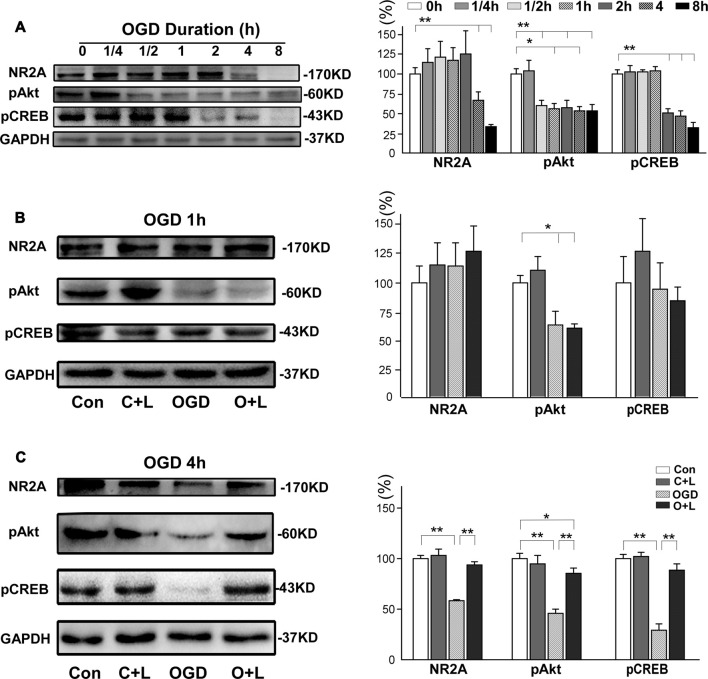
Effects of LBP on the expression of NR2A, pAkt and pCREB proteins after exposed 4 h OGD. **(A)** Expression of these proteins at different OGD duration. **(B)** When exposed to OGD for 1 h, LBP did not influence expression of NR2A, pAkt and pCREB proteins. However, LBP treatment markedly inhibited the decrease in these protein levels after neurons were exposed to 4 h OGD (**C**; *P < 0.05; **P < 0.01).

Next, we investigated whether LBP-induced neuroprotection against OGD was also mediated by the NR2A-Akt-CREB pathway. Our findings demonstrated NR2A expression was maintained at normal levels with 2 h of OGD exposure before it decreased to 66.5 ± 11.4% at 4 h OGD and 33.5 ± 2.8% at 8 h OGD, respectively (both *p* < 0.01 vs. control, Figure 6A). Expression of pAkt dramatically decreased from OGD for 30 min to OGD for 8 h (59.5% ± 7.1, 55.9% ± 7.1, 57.5% ± 9.6, 53.6% ± 4.6 and 52.9% ± 8.4, respectively; all *p* < 0.01 vs. control). Expression of pCREB remained unchanged with 1 h OGD, but dramatically decreased to 50.8% ± 4.6 at 2 h OGD, 46.2% ± 7.4 at 4 h OGD and 32.1% ± 6.6 at 8 h OGD (all *p* < 0.01 vs. control). We further investigated the effects of LBP on the expression of major proteins related NR2A signaling. At 1 h OGD exposure, we observed no differences in expression of NR2A, pAkt or pCREB between the OGD group and OGD+LBP group (*p* > 0.05, Figure 6B). However, at 4 h OGD exposure, LBP significantly reversed the decrease in expression of NR2A (from 58.6% ± 0.7 to 93.8% ± 2.0), pAkt (from 45.7 ± 2.4% to 85.9 ± 2.9%) and pCREB (from 29.5% ± 3.2 to 88.5% ± 3.4), respectively (all *p* < 0.01), when compared with the OGD group (Figure 6C). LBP upregulated the NR2A and pCREB expression to the control level (*p* > 0.05 vs. control), although expression of pAkt remained lower than the control (*p* < 0.05 vs. control).

### LBP Diminished the Effects of NR2A Antagonist and NR2B Co-Agonist during OGD

**Figure 7:**
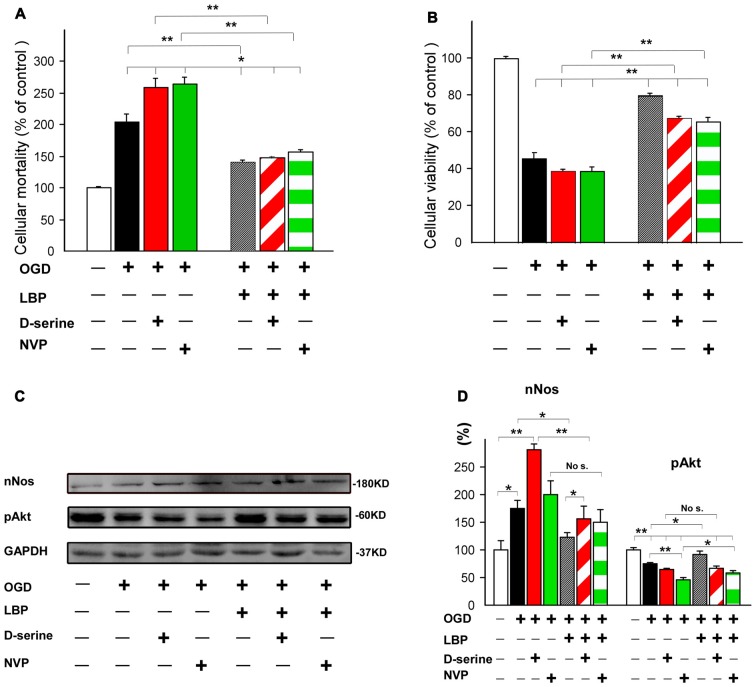
Neuroprotective effect of LBP was confirmed by using NR2B agonist D-serine and NR2A antagonist NVP-AAM077. LBP treatment dramatically antagonized the increase in cellular mortality caused by D-serine and NVP-AAM077 **(A)** and the decrease in cellular viability in response to 4 h OGD **(B)**. Furthermore, LBP primarily blocked the increase in expression of nNOS caused by D-serine and inhibited the decrease in expression of pAkt caused by NVP-AAM077 when cultured neurons were exposed to OGD for 4 h (**C,D**; *P < 0.05; **P < 0.01).

To further confirm that LBP-induced neuroprotection was mediated by inhibiting NR2B signaling or activating the NR2A signaling pathway, we employed a NR2B co-agonist (D-serine) and a NR2A antagonist (NVP-AAM077) in this study. Our results showed that 4 h OGD caused twice the neuronal mortality (205.1% ± 12.6, *p* < 0.01 vs. control; Figure 7A). This mortality was further enhanced by administration of D-serine (259.1% ± 14.6) or NVP-AAM077 (264.5% ± 10.6; all *p* < 0.05 vs. OGD alone). LBP treatment markedly blocked the increased cell mortality caused by D-serine or NVP-AAM077 and reduced neural death to 140.8% ± 2.3 in the LBP+D-serine group or 156.4% ± 4.5 in the LBP+NVP-AAM077 group (*p* < 0.01 vs. D-serine or NVP-AAM077 group, respectively). Consistent with the observed neuron death results, cell viability decreased to 45.9% ± 9.7 in the OGD group (Figure 7B), which was further reduced by application of D-serine (38.8% ± 1.3) or NVP-AAM077 (38.6% ± 2.4). However, LBP treatment considerably prohibited reduced neuronal viability induced by D-serine or NVP-AAM077 and increased neural viability to 67.4% ± 1.5 in the LBP+D-serine group and 65.7% ± 2.6 in the LBP+NVP-AAM077 group (*p* < 0.01 vs. D-serine or NVP-AAM077 group, respectively). These results indicated that LBP’s neuroprotection was mediated by its dual roles in blocking NR2B signaling and activating NR2A signaling. To further confirm this conclusion, we determined the effects of D-serine or NVP-AAM077 on expression of nNOS (a major protein of NR2B signaling pathway) and pAkt (a major protein of NR2A signaling pathway). As shown in Figures 7C,D, the nNOS expression increased to 174.0% ± 15.2 after 4 h OGD exposure, and this expression was further enhanced to 280.3 ± 11.9% in the presence of D-serine (*p* < 0.01 between OGD alone group and D-serine group). However, LBP treatment dramatically blocked the expression of nNOS to 122.5% ± 9.0 (*p* < 0.01 vs. D-serine group). LBP+NVP-AAM077 group invariably showed a slight reduction of nNOS expression compared with NVP-AAM007 alone group, although not statistically significant. These results suggest that LBP reduced expression of nNOS through inhibiting NR2B signaling pathway. Furthermore, pAkt expression significantly decreased in response to OGD alone (75.3% ± 1.4, *p* < 0.01 vs. control), which was further downregulated with administration of NVP-AAM077 to 46.4% ± 3.3 (*p* < 0.01 vs. OGD alone group). However, LBP reduced the inhibition of NVP-AAM077 and increased pAkt expression to 58.8% ± 3 (*p* < 0.05, compared between NVP-AAM077 group and LBP- NVP-AAM077 group). There was no significant difference of pAkt expression between D-serine group (64.2% ± 2.3) and LBP+D-serine group (67.1% ± 4.5, *p* > 0.05). Taken together, these results indicated that LBP upregulates expression of pAkt by stimulating NR2A signaling pathway as well as downregulates nNOS expression by inhibiting NR2B signaling pathway after OGD.

## Discussion

**Figure 8:**
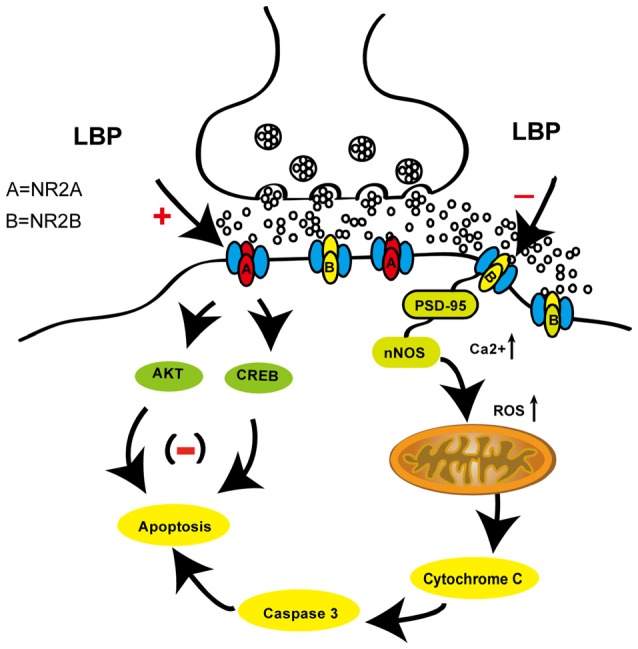
A schematic picture showing the neuroprotective effects of LBP through inhibiting the NR2B signaling pathway and activating the NR2A signaling pathway against ischemic/hypoxia injury.

In the present study, our results demonstrated that LBP significantly prevented CA1 neuronal damage after transient global ischemia and reduced cortical neuronal death in response to OGD *in vitro*. LBP also ameliorated memory deficits after ischemia. Here, we provide the first evidence indicating that the neuroprotection of LBP is mediated by inhibiting the expression of major proteins in the NR2B signaling pathway including NR2B, nNOS, Bad, cytC and cleaved caspase-3, mitochondrial stress and calcium influx (Figure 8). LBP also prevented the reduction in expression of major proteins of NR2A signaling pathway including NR2A, pAkt and pCREB.

Strong evidence has shown that LBP exhibits neuroprotective effects through anti-oxidative stress pathways or by inhibiting JNK signaling in different injury models (Li et al., 2013; Xing et al., 2016). In ischemic models, LBP reduced infarct area and ameliorated neurological dysfunction in MCAO mice through anti-apoptotic mechanisms and protection of blood brain barrier (Yang et al., 2012; Wang T. et al., 2014). Although it has been reported that LBP can antagonizes glutamate toxicity on neurons through the JNK pathway (Ho et al., 2009), here we first reported that LBP exerts its neuroprotective effect by modulating both NR2A and NR2B expression and their signaling pathways. The regulation on these two pathways by LBP is not likely to occur at mild OGD when their protein expression is stable, but LBP exerts its effect during severe OGD/ischemia which causes changes in expression of proteins in these pathways. During mild injury (1 h OGD), expression of PSD95, Bad, cytC and cleaved caspase-3 in the NR2B signaling pathway did not change while NR2B and nNOS levels increased. Interestingly, administration of LBP only reduced expression of NR2B and nNOS but did not affect expression of other proteins in the pathway. It is currently unknown on the underlying mechanisms. It will be interesting to further investigate whether LBP regulates protein turnover of NR2B and nNOS through post-translational modification or directly regulates transcription of these genes. Concerning NR2A signaling in response to mild OGD, LBP application did not modify NR2A and pCREB levels as indicated by lack of alteration in their expression. As for pAkt expression, administration of LBP for 1 h under OGD conditions could not upregulate its expression when it was reduced at this time point. It has been reported that NMDA induces maximal activation of Akt phosphorylation at 3 h (Zhu et al., 2002). Therefore, we speculate that LBP upregulates expression of pAkt by increasing the protein expression of NR2A. This is supported by our observation that when exposed to severe injury (4 h OGD), LBP treatment significantly increased expression of NR2A, pCREB and pAkt when the expression of these proteins was decreased. LBP treatment also markedly blocked the increase in expression of major proteins in the NR2B signaling pathway such as nNOS, Bad, cytC and c-caspase-3 under 4 h OGD conditions. We cannot rule out the possibility that LBP can reduce NR2B expression under this condition. However, when NR2B level has already been markedly reduced during this period, LBP did not further inhibit NR2B expression. The reduced expression of NR2B at 4 h and 8 h OGD may be caused by a compensatory mechanism responding to the increased level of NR2B at early stage of OGD (1 h). Therefore, the inhibited effect of LBP on NR2B expression mainly occurred in the early stage of OGD.

It is plausible that in the early stage (1 h OGD), overexpression of NR2B and nNOS may induce the formation of a NR2B-PSD-95-nNOS complex. This complex will trigger production of NO followed by apoptosis (Christopherson et al., 1999; Sattler et al., 1999). Therefore, LBP may block the formation of the NR2B-PSD-95-nNOS complex by inhibiting expression of NR2B at the time point. At late stages (OGD for 4 h), however, when expression of NR2B was completely depressed, LBP did not further inhibit NR2B expression. LBP still prohibited overexpression of nNOS, which may be mediated through inhibiting a cascade of events related to mitochondrial injury and apoptosis, including reduction of calcium influx, mitochondrial permeability, Bad, cytC and caspase-3 levels after 4 h OGD. This is consistent with prior finding in an *in vivo* MCAO model (Wang T. et al., 2014). Finally, we used a NR2A inhibitor NVP-AAM077 and NR2B co-agonist D-serine to test whether the neuroprotective influence of LBP would antagonize the effect of these two drugs. Our results indicate that LBP can greatly block the action of NVP-AAM077 and D-serine, which suggests that LBP imparts its neuroprotective effects through inhibiting NR2B or activating NR2A.

Interestingly, LBP plays its dual roles in acting as an antagonist to NR2B while as an agonist to NR2A under ischemic/hypoxic conditions. This may stem from the presence of multiple components of LBP with structures composing of different backbones and branches (Wang et al., 2009; Zou et al., 2010; Wang Z. et al., 2014; Yuan et al., 2016). These multiple components, together with other ligands including glycine and glutamate, may exert complex effects on NMDA receptors. Furthermore, the different synaptic localization of NR2A (intrasynaptic)- and NR2B (extrasynaptic)-containing NMDA receptors may account for the dual roles of LBP as observed in this study. Therefore, LBP can be the regulator for both blocking NR2B expression and upregulating NR2A expression under hypoxic/ischemic conditions. It has also been reported that, LBP can protect neurons in the optic nerve injury model by inhibition of JNK pathways and scavenging free radical (Lin et al., 2009; Li et al., 2013). Thus, LBP exerts neuroprotective effects through multiple pathways. Further studies are needed to dissect the exact mechanisms by which LBP exerts under hypoxic/ischemic conditions.

It should be noted that for our *in vitro* analyses, we set a control-LBP group to investigate whether LBP affects neural viability and expression of major proteins in the NR2B and NR2A signaling pathways under normal physiological conditions. Our results indicated that there were no significant changes by LBP treatment, suggesting that LBP is safe to neuronal cells. Overall, the present study demonstrated that LBP is neuroprotective against ischemic injury by its dual roles in activation of NR2A-mediated survival pathway and inhibition of NR2B-mediated apoptotic pathway, which suggests that LBP may be a superior therapeutic candidate for the treatment of ischemic stroke.

## Author Contributions

YR and KS designed and organized the study, revised and approved the manuscript. ZS and LZh performed experiments, data analysis and wrote the manuscript. TL, LZe, YX, XH, LX, XT, JN, YH and ZL performed experiments, collected and analyzed data. ZX, YW and CKT revised and approved the manuscript.

## Conflict of Interest Statement

The authors declare that the research was conducted in the absence of any commercial or financial relationships that could be construed as a potential conflict of interest.
